# *Cheilospirura hamulosa* (Nematoda: Acuariidae) infection in indigenous chickens in Bangladesh: epidemiology, pathology and anthelmintic efficacy

**DOI:** 10.1017/S0031182025000319

**Published:** 2025-04

**Authors:** Akter Shirin, Nusrat Nadia, Md. Shahadat Hossain, Sharmin Shahid Labony, Sumaya Naznin Ritu, Md. Haydar Ali, Umme Razia Islam, Romana Parvin, Sadia Afroz Esha, Anita Rani Dey, Md. Mahmudul Alam, Mohammad Zahangir Alam, Md. Abdul Alim, Anisuzzaman

**Affiliations:** 1 Department of Parasitology, Bangladesh Agricultural University, Mymensingh, Bangladesh; 2 Department of Pathology and Parasitology, Hajee Mohammad Danesh Science and Technology University, Dinajpur, Bangladesh; 3 Department of Surgery and Theriogenology, Bangladesh Agricultural University, Mymensingh, Bangladesh

**Keywords:** anthelmintics, *Cheilospirura hamulosa*, eosinophils, gizzard, inflammation

## Abstract

Helminth infection is highly prevalent in indigenous chickens reared in semi-scavenging/ scavenging systems in Bangladesh. Here, we estimated the prevalence of gizzard worm infection in indigenous chickens, the detection of the worm-induced pathologies, the development of *ex vivo* cultural protocol, and anthelmintic efficacy. We randomly collected and examined 390 chickens and isolated worms from the gizzard and proventriculus. The isolated worms were identified as *Cheilospirura hamulosa* Diesing, 1861. The overall prevalence of *C. hamulosa* was 33.1% (129 out of 390). Prevalence of the worm was almost similar in both sexes but significantly (p <0.05) higher in adult chickens (44.3%) and in the summer season (47.1%). In heavy infections, *C. hamulosa* destroyed the muscular layer of the gizzard. The presence of brown necrotic tissues and curd-like caseous materials was detected in the affected gizzards. In severe cases, the horny lining of the gizzard was inflamed, necrotized and marked by multiple holes and brick-red colored spots. Liquefied, fetid materials oozed out from the muscular layer in extensive cases. Histopathological examination showed marked infiltrations of eosinophils. In serum-supplemented M199 and DMEM, adult *C. hamulosa* survived well and reproduced. Levamisole (LEV) and ivermectin (IVM) efficiently killed the worm. However, albendazole (ABZ), mebendazole (MBZ) and piperazine (PPZ) did not kill the worms. Our results suggest that *C. hamulosa* is highly prevalent in semi-scavenging chickens in Bangladesh. LEV and IVM can be used to treat and control the infection in chickens.

## Graphical abstract

**Figure:**
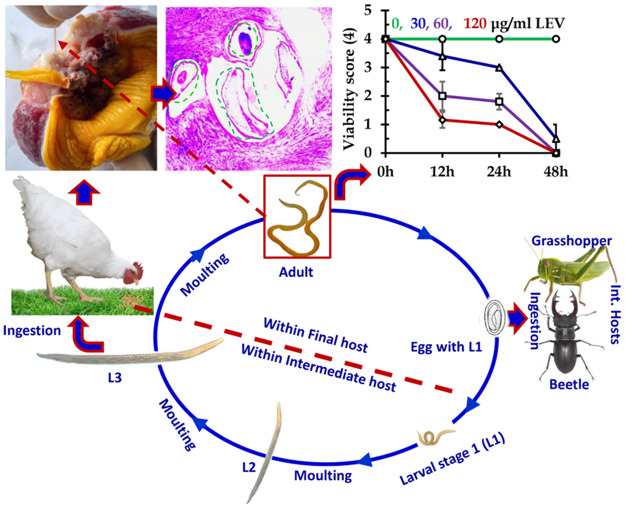

## Introduction

Poultry refers to domesticated birds reared to obtain valuable products like meat, eggs and feathers. Poultry farming is very popular and directly linked to several goals (e.g. Goal 1 − 5, Goal 8, Goal 10 and Goal 12) of Sustainable Development Goals (SDGs). As it is the cheapest source of high-quality animal protein, a lower middle-income country like Bangladesh largely depends on poultry and poultry products to fulfill daily protein requirements. Although there is a vast expansion of commercial poultry farms in Bangladesh, indigenous chickens (ICs) are the most famous, and their market value is very high. ICs (*Gallus gallus domesticus*) are specially adapted to harsh environmental conditions, particularly in small-scale, free-range and organic production systems (Padhi, 2016). ICs in scavenging and semi-scavenging systems frequently harbor endoparasites affecting the intestine, gizzard, caeca, liver and reproductive tract (Rabbi et al., 2008; Labony et al., 2022; Shohana et al., 2023; Ritu et al., 2024). Several species of gizzard worms infect chickens, but the most prevalent species is *Cheilospirura hamulosa* (Nematoda: Acuariidae). Its prevalence may become as high as 45% in chickens (Graves and Fedynich, 2013). The worm can also infect other birds; for example, *C. hamulosa* has also been isolated from pheasants (14.3%) in Brazil (Menezes et al., 2003).

*C. hamulosa* has an indirect lifecycle, and grasshoppers and beetles act as intermediate hosts. Chickens become infected by eating grasshoppers and beetles containing viable infective larvae (L3). Semi-scavenging poultry primarily collect their food from the environment. They are fond of insects like grasshoppers and beetles. Therefore, the chances of being infected with *C. hamulosa* are very high whileroaming backyard. During scavenging, they ingest arthropods, including, grasshoppers and beetles, containing L3 larvae of the worm, and get infected. *C. hamulosa* is typically located beneath the horny layer of the gizzard covering the inner muscular layer of the gizzard and significantly hampering its function. The symptoms of the infection may vary; however, severe infections cause anemia, emaciation, droppings of wings and weakness (Salam et al., 2009).

Anthelmintic vaccines aiming to combat poultry helminth are yet to be developed (Anisuzzaman and Tsuji, 2020). Therefore, treatment and mitigation programs targeting helminth infections, including *C. hamulosa*, rely mainly on prophylactic chemotherapy. In our country, commonly used drugs include albendazole (ABZ), fenbendazole (FBZ), mebendazole(MBZ), piperazine (PPZ), levamisole (LVZ) and ivermectin (IVM). Our group recently detected benzimidazole and PPZ resistance in *Ascaridia galli* (Ritu et al., 2024). We found no information from online-based literature on the efficacy of commercially available anthelmintic against *C. hamulosa*. Furthermore, the impacts and detailed pathologies induced by the worm are yet to be studied. Therefore, the present study was conducted to determine the prevalence of the infection and pathologies induced by the worm in ICs in Bangladesh and to determine the efficacy of commercially available anthelmintics against *C. hamulosa*.

## Materials and methods

### Study area

The study was conducted in the Mymensingh division (the middle part of the country located between 24°38ʹ and 24°54ʹ north latitudes and between 90°11ʹ and 90°30ʹ east longitudes.

### Sample size

The sample size was calculated using the formula *n* = *Z*2*P* (1 − P) \*d*2 where *n* is the sample size, *Z* = 95%, statistics for confidence level, *P* = 50%, expected prevalence; and *d* = 0.05 is the precision (Daniel and Cross, 2019). Using this formula, the calculated sample size was 385. However, 390 chickens of both sexes and different age groups were collected and examined for the research. The chickens were divided into two major groups based on their age. Such as adult (≥6 months of age) and young (<6 months of age).

### Study period and data recording

The study was conducted for 3 years, from 2020 to December 2023. While purchasing chickens, some data regarding age and sex were recorded by asking the poultry owner. To evaluate the effect of seasons, each year was divided into three major seasons: summer (March–June), monsoon (July–October) and winter (November–February) seasons.

### Post-mortem examination

Chickens were sacrificed by cutting with carotid arteries following the halal Muslim method, and subjected to routine post-mortem examination. Abdominal contents were removed. The serosal surface of the gizzard and proventriculus was separated and examined carefully before opening.

### Collection and identification of C. Hamulosa

After external examination, both the gizzard and proventriculus were opened. Then, the internal surface of the organs was examined closely to detect red or brown circumscribed lesions embedded with worms. The horny layer of the gizzard was removed carefully to detect and collect parasites. Worms were collected from the gizzard with forceps and placed into a petri dish containing normal saline.

### Determination of histopathological changes

During the post-mortem examination, affected or suspected samples were collected and preserved in Corony’s solution (a mixture of absolute ethanol and glacial acetic acid at ratio 3:1) for 48 h under gentle shaking. Then, samples were washed with the PBS, and thin (5 μm) sections were made. Sections were then stained with H&E and examined under a light microscope in a blinded manner. At least three sections of each tissue sample and three foci were examined at 10x and 40x objectives.

### Collection of the serum

Helminth infection-free ICs (*Gallus gallus domesticus*) and khaki Campbell (*Anas platyrhynchos*) ducks of 6 months of age and both sexes were considered for blood collection. Blood was collected from the wing vein into non-medicated and sterile tubes. Blood samples were kept at room temperature for 30 min and centrifuged at 8500***g*** for 10 min, and serum was collected. The collected serum was filtered through a syringe filter having 0.2 μm pore size (Minisart, Sartorius Stedim Biotech, Vienna, Austria) and preserved at −20 °C. Before use, the serum was thawed at room temperature and heated at 56 °C for 30 min.

### Ex vivo culture of C. Hamulosa

After isolation, the worms were washed in sterile PBS containing 200 U/ml of penicillin and 200 μg/ml of streptomycin and transferred into M199 (Hyclone, Utah, USA), DMEM (Hyclone, USA), or RPMI 1640 (Hyclone, USA), Adult *C. hamulosa* (1 parasite/well in a final volume of 2 ml) was cultured in Medium M199, DMEM, or RPMI 1640 added with either fetal calf serum (FCS, Hyclone, USA), chicken serum (CS), or duck serum (DS) at different concentrations (5− 20%). The parasites were incubated in a 24-well flat bottom tissue culture plate at 37 °C with 5% CO_2_ in a humidified chamber for up to a week. Experiments were conducted in triplicates for each condition, with medium replacement every alternative day. Viability screening (0-4) was performed based on the tegument’s sharpness, the parasites’ movement, their pharyngeal pulp, and tail movement, as described previously (Ritu et al., 2024).

### Determination of the efficacy of the anthelmintic

Adult *C. hamulosa* was collected, washed and cultured in DMEM supplemented with 20% CS coupled with penicillin 200 U/ml and streptomycin 200 μg/ml, following the condition mentioned earlier and maintained overnight. Then, ABZ, LVZ, MBZ, IVM and PPZ were added at different concentrations. ABZ, MBZ and LVZ were added at 20−120 µg/ml and IVM at the concentration of 0.5−2 µg/ml. PPZ was used at a concentration of 100−500 µg/ml. The cultured worms were examined at different time points (0−96 h). Experiments were conducted in triplicates for each condition. Non-treatment *C. hamulosa* served as controls.

### Statistical analysis

The generated data were plotted into a Microsoft Excel spreadsheet. Descriptive statistics were employed to analyze the data obtained in this study. Epidemiological data was analyzed using the *Z* test, while data related to anthelmintic efficacy were subjected to one-way ANOVA, followed by Bonferroni post-hoc analysis.

## Results

### The overall prevalence and morphological features of the identified C. Hamulosa

**Figure 1.**
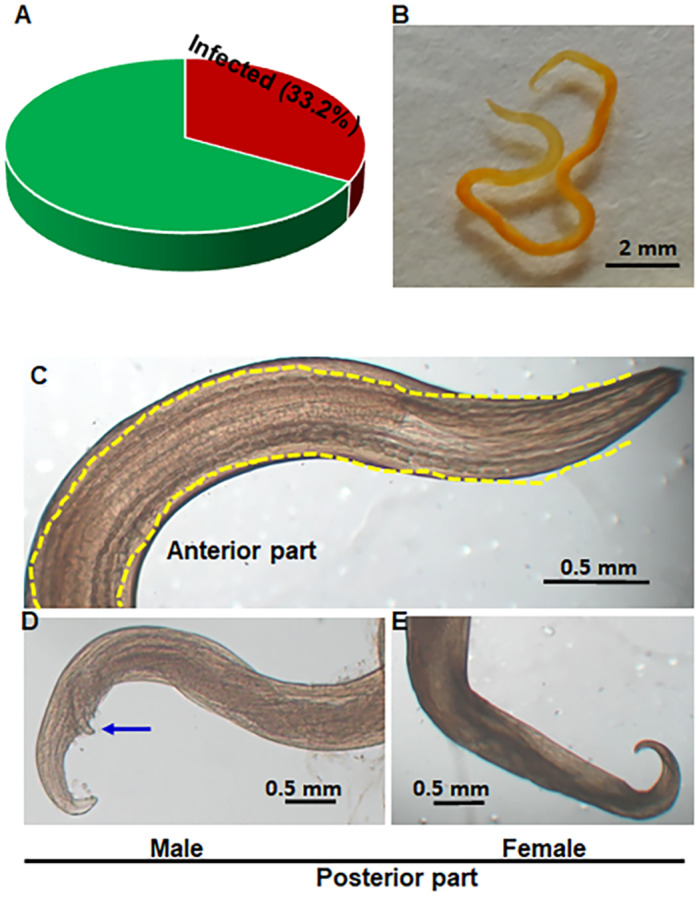
Overall prevalence and morphological features of *Cheilospirura hamulosa.* (A) Overall prevalence of *C. Hamulosa* in chickens. (B) Adult parasite (whole mount). (C) Anterior part of the worm. Dotted yellow lines are cordons. (D) Posterior part of the male. Blue arrow indicates spicule. (E) Posterior part of the female.

Of the examined chickens, 33.1%, (129 out of 390) were infected with *C. hamulosa* (Figure 1A). During detailed morphological and morphometrical analysis, the isolated worms were short, thin, slender and reddish to brown in colour. Female worms were longer than males (Figure 1B). The length of the adult females varied from 16 to 29 mm (18.5 ± 2.4 mm), but that of males were 10−14 mm (12.3 ± 1.2 mm) long. Both males and females were straight, displaying a moderate and consistent thickness throughout the body with tapering ends. The anterior end of the worm featured a buccal capsule, a specialized structure for attaching to the mucosal layer of the host. The characteristic digitiform tail was observed in females. On the contrary, unequal spicules, caudal alae and ten pairs of caudal papillae were seen in males. All the worms were especially characterized by cordons, double cuticular ridges with an irregular outline (Figure 1C). The characteristic longitudinal cordons and muscular and glandular esophagus were observed in both sexes. The ratio between the cordons and body length in males and females was 1: 1.33 and 1: 1.68, respectively. The ratio between long and short spicules in males was 1: 2.3, confirming the worms as *C. hamulosa* (Figure 1D, E).

### Associated factors governing the prevalence of worms

**Figure 2.**
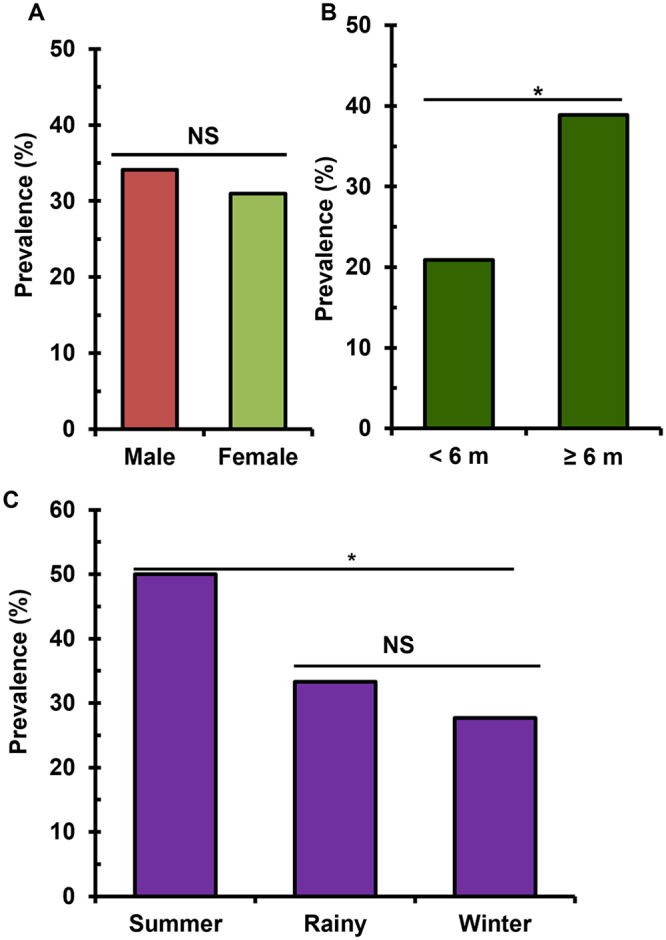
Factors associated with *Cheilospirurahamulosa* infections. (A) Sex-wise prevalence, (B) age-wise prevalence, and (C) season-wise prevalence of *C. Hamulosa*. M, months.

To evaluate the impact of the host’s sex on the prevalence of the infection, a comparable number of chickens of both sexes (male, *n* = 196 and female, *n* = 194) were collected and examined. The results showed that the sex of the host did not have any significant impact on the prevalence of *C. hamulosa* infection. However, the prevalence of the worm was a bit (*p* > 0.05) higher in males (34.2%, 67 out of 196) than in females (31.9%, 62 out of 194) (Figure 2A). To assess the influence of the age of chickens on the prevalence of the worm, the examined chickens were categorized into two age groups: young (*n* = 205) and adult (*n* = 185). We observed that the prevalence of *C. hamulosa* infection was significantly (*p*<0.05) lower (20.5%, 38 out of 185) in young chickens than in adult (44.3%, 91 out of 205) chickens (Figure 2B). On the other hand, we examined a comparable number of chickens in the summer (*n* = 138), rainy (*n* = 139) and winter (*n* = 113). The study revealed that the prevalence of *C. hamulosa* was significantly (P < 0.05) higher in the summer season (47.1%, 65 out of 138) than in the rainy (30.9%, 43 out 139) and winter (18.6%, 21 out of 113) seasons (Figure 2C).

### Gross pathologies induced by C. hamulosa infection

**Figure 3.**
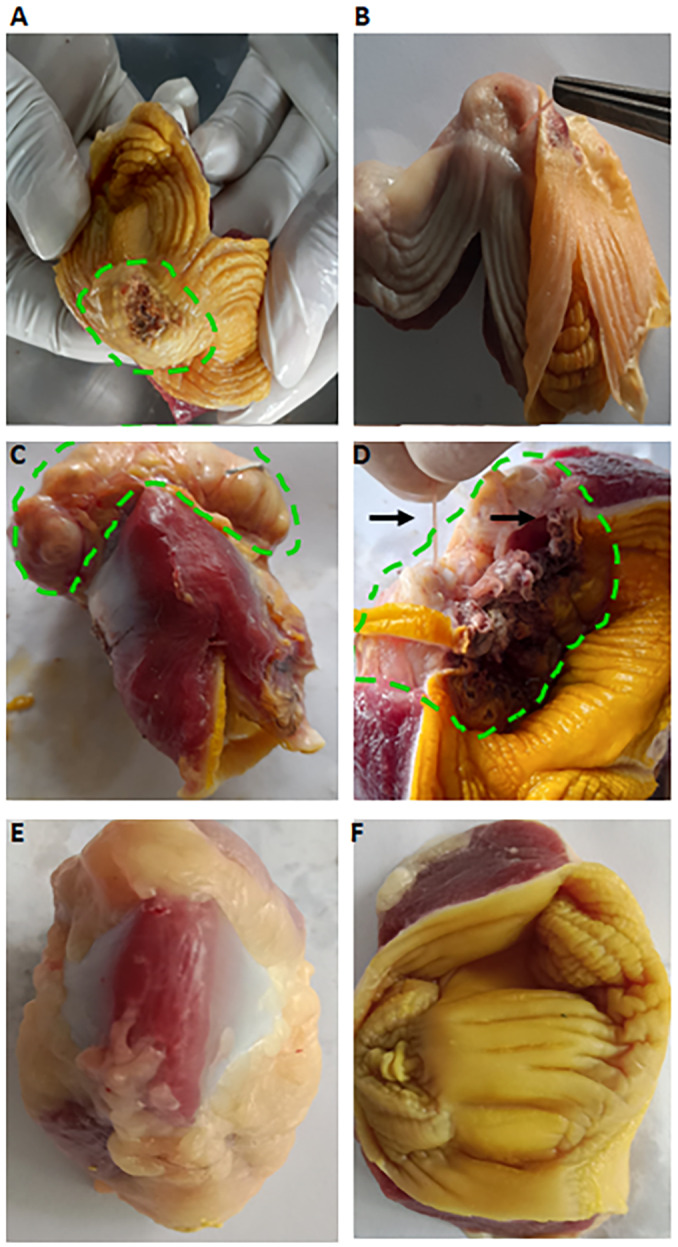
Gross pathological changes induced by *Cheilospirura hamulosa*. (A) Mild infection with *C. Hamulosa*. Dotted circular line indicates small lesion. (B) Adult *C. Hamulosa* (black arrow) attached to the muscular wall of the same gizzard after removal of koilin lining. (C) Heavy infection with the worm. Development of sac-like or tumor-like growth. Green dotted line indicates the tumor-like growth of the gizzard. (D) Inner surface of the same gizzard showing severe damage of the muscular wall and the necrotized koilin lining. (E) Serosal surface of normal gizzard of age and sex-matched ICs. (F) Internal surface of the same gizzard.

The worms were mainly detected under the horny layer of the gizzard. However, *C. hamulosa* was also detected at the junction of proventriculus and the gizzard in a few cases. Indeed, severity of the lesions depends on the burden of the worms. Within low parasitic burden, only petechial hemorrhagic spots were detected in the Koilin membrane, the lining of the internal wall of the gizzard. In the mild infection, the Koilin membrane displayed small, dark red, ulcerated areas with soft yellowish-red nodules. In severe infections, widespread ulcerations were detected. Gizzards sometimes became so weak that a sac-like structure was developed. Brown necrotic tissues and curd-like caseous materials were detected (Figure 3A, B). In heavy infection, the entire layer of the musculature of the gizzard was found to be affected. The horny lining of the gizzard was inflamed, necrotized and marked by multiple holes and brick-red spots. The horny layer became loose or detached from the muscular layer of the gizzard (Figure 3C, D). On the cut surface, rotten caseous materials oozed out from the muscular wall of the gizzard. Such changes were not detected in the age- and sex-matched gizzard of ICs (Figure 3E, F).

### Histopathological changes evident in gizzards

**Figure 4.**
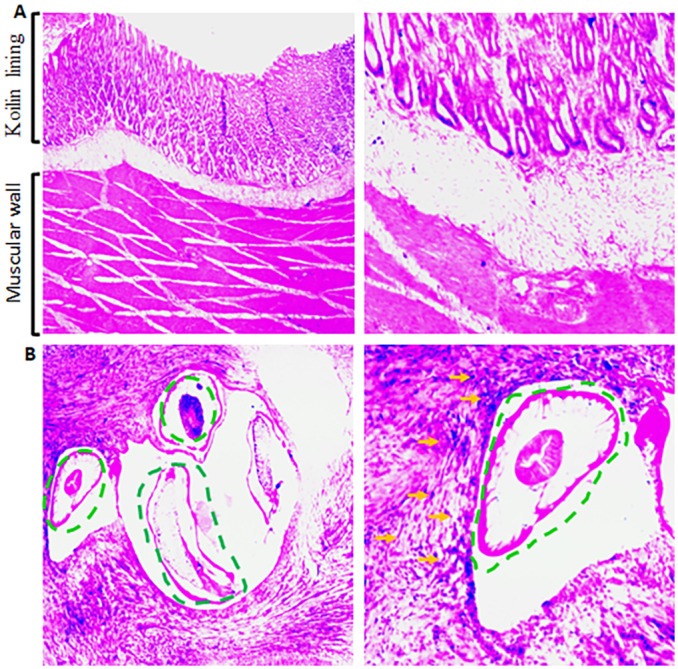
Histopathological changes induced by *Cheilospirura hamulosa*. (A) Histological sections of the age and sex-matched indigenous chickens (ICs) as mentioned in Figure 3E, F. Left panel is at low magnification (10x objective). Right panel is at higher magnification (40x objective). (B) Histological sections of the age- and sex-matched ICs as mentioned in Figure 3C, D. Left panel is at low magnification (10x objective). Dotted lines indicate cross sections of the parasite. Right panel is at higher magnification (40x objective). Dotted lines indicate cross sections of the parasite. Green arrows indicate inflammatory cells.

Histological sections stained with H&E showed that the normal gizzards had well-defined layers of Koilin membrane and the muscular wall of the gizzards (Figure 4A). However, histological analysis of the *C. hamulosa*-infected gizzards of age- and sex-matched ICs by independent pathologists revealed a massive proliferation of fibrous connective tissues in the muscular wall of the gizzard. Cross sections of the parasite were detected in the histological section of the wall of the gizzard. In most of the cases, infiltration of eosinophils, heterophils and mononuclear cells was detected. The development of micro-granuloma was also evident (Figure 4B).

### Survival and reproduction of the worm in ex vivo culture platform

**Figure 5.**
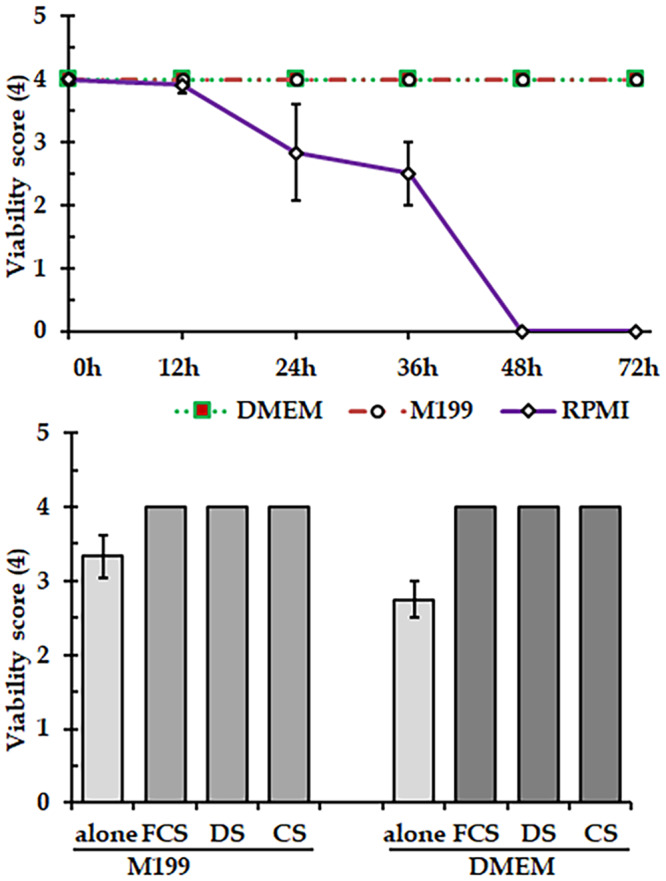
Development of an *ex vivo* culture protocol for *Cheilospirura hamulosa.* (A) Selection of media for the *ex vivo* culture of *C. Hamulosa*. (B) Viability of *C. Hamulosa* after addition of nutrient supplements. FCS, fetal calf serum; DS, duck serum; CS, chicken serum.

To select suitable commercial media, adult *C. hamulosa* was cultured in various commercially available media (e.g. RPMI, M199 and DMEM) up to 7 days and assessed at different time points. We found that the viability of the worms decreased rapidly in RPMI within 48 h, which declined to a score of 0.5 (out of 4). In RPMI, all worms died within 72 h. In contrast, DMEM and M199 efficiently supported the worm’s survival and reproduction, maintaining viability scores above 3.5 even after 72 h (Figure 5A). Then, the viability of the parasites declined gradually, and all parasites died by day 7 (viability score 0). Therefore, we decided to use either DMEM or M199 for the *ex vivo* culture of *C. hamulosa*. In the next, addition of FCS, CS or DS in DMEM or M199 significantly increased the worms’ viability and supported *C. hamulosa* to survive up to 7 days with an average viability score of 4. Since FCS is costly, and chickens are the principal natural hosts of *C. hamulosa*, CS-supplemented DMEM media were used for the culturing of the worm. And, this protocol was used for screening the efficacy of commercially available anthelmintics (Figure 5B).

### Efficacy of commercially available anthelmintics

**Figure 6.**
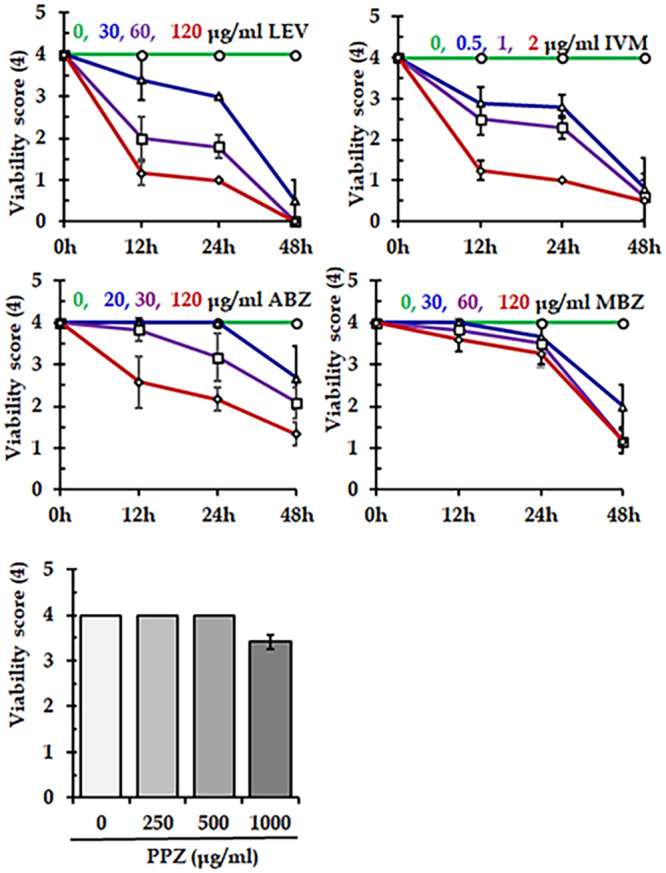
Efficacy of different commercially available anthelmintics against *Cheilospirura hamulosa.* LEV, levamisole; IVM; ivermectin; ABZ, albendazole; MBZ, mebedazole; PPZ, piperazine.

Using long-term *ex vivo* culture protocol, we tasted the efficacy of several commonly used anthelmintics. We found that LEV and IVM significantly (*p* < 0.05) reduced the viability score of the worm in a concentration-dependent manner. At 120 μg/ml concentration, LEV notably decreased the viability score within 12 h and completely killed all *C. hamulosa* within 48 h post-treatment (p.t). Also, IVM, at 2 μg/ml concentration, significantly (*p* < 0.05) decreased the viability score within 12 h and reduced the viability score to 1.5 by 24 h p.t. In contrast, at the highest recommended concentration (120 μg/ml), ABZ and MBZ failed to kill the worm even after 48 h, indicating that ABZ and MBZ were ineffective against *C. hamulosa* infections in Bangladesh. Furthermore, PPZ, at 500 µg/ml concentration, did not kill the worm after 48 h (Figure 6).

## Discussion

Chickens were domesticated > 8000 years ago in Southeast Asia and were introduced to other parts of the world by sailors and traders (Lawal et al., 2020). Nowadays, chickens are the most important poultry species on the globe. According to recent estimates, chickens provide 39.57% of animal proteins (138.75 out of 350.64 metric tons) globally. There are about 33 billion domestic chickens worldwide (Shahbandeh, 2024). And in Bangladesh, the chicken population reached 319.69 million (Shamim, 2024). It not only meets protein demand but also provides employment opportunities for people. ICs are adapted to harsh environmental conditions, particularly on a small scale, and are reared in free-range and organic production systems. Although the prevalence of *C. hamulosa* infection is very high in scavenging or semi-scavenging chickens, very few studies have been conducted globally only to report the prevalence and gross pathological changes induced by the worm (Katoch et al., 2012; Alam et al., 2014). Here, we provide information regarding the present status, morphology, gross and histopathology of affected tissues, cultural protocol of the worm, and the efficacy of commercially available common anthelmintics.

The present study revealed that 33.1% of ICs were affected with *C. hamulosa*. The worm is the least studied globally compared to *Ascaridia galli* and also in Bangladesh. A previous study reported that only 8.75% of ICs were infected with the worm in Bangladesh (Alam et al., 2014). However, the research group examined only young chickens 2−4 months of age. In the contrary, we examined chickens of all ages, and *C. hamulosa* are less prevalent in young chickens. Therefore, the very low prevalence of the worm recorded in the previous study is not surprising. These worms have also been studied in other countries. Katoch et al. (2012) reported a very high prevalence (72%) of gastrointestinal helminth, including gizzard worms in scavenging chickens from India. Similarly, two decades ago, Nowicki et al. (1995) reported a very high prevalence (98%) of the gizzard worm infection in Canada geese (*Branta canadensis*) in the USA. The worm is distributed globally, covering almost all climatic zones. The nematode has also been detected in European countries. In France, 67% of chickens were affected with worms (Fanelli et al., 2020). In Ghana, the prevalence of gizzard worms in scavenging chickens is 25% (Poulsen et al., 2000). In central Patagonia, Argentina, the prevalence of gizzard worms detected in anatid ducks was 77% (Agüero et al., 2016). The difference among the results may vary due to the variation in the geographical location of the research area, method of detection, sample size and host species.

We found that the age of ICs, but not sex, had an impact on the prevalence of the worm. A higher prevalence of infection was found in adult chickens (>6 months of age), indicating that adults were more likely to get infection than their younger counterparts. We presumed that several factors contributed to this observed difference, including the potential accumulative exposure over time, feeding and differences in behavior or environmental interactions with the two age groups. The lifecycle of *C. hamulosa* is indirect. Various arthropods, such as beetles and grasshoppers, act as intermediate hosts. Young chickens have very limited roaming tendencies. They mostly live on feeds supplied by the owners and rarely scavenge from nature. This feeding behavior of younger chickens possibly greatly influences the lower prevalence of the worm in younger birds.

Estimation of the season-wise prevalence of *C. hamulosa* infection revealed that the prevalence of the worm was significantly (*p* < 0.05) higher in the summer season than in the rainy and winter seasons. The elevated prevalence of worms in summer could be attributed to environmental factors. In summer, specific conditions such as temperature, humidity and ecological changes may contribute to this significant seasonal variation in infection rates. In addition, in summer seasons (March−June), there are the highest populations of arthropods, including grasshoppers and beetles in Bangladesh, which act as an important intermediate hosts of *C. hamulosa*. Possibly, the higher availability of the intermediate hosts in the summer season in Bangladesh largely contributes to the higher prevalence of the worm in that season.

This study suggests that *C. hamulosa* can cause various pathological lesions in chickens, ranging from a few small, dark-red, ulcerated areas with soft yellowish-red nodules to hemorrhagic changes in gizzards. Similar types of lesions were recorded by Menezes et al. (2003). In some cases, we found necrotic areas with cellular infiltrations, which conform the findings of Oryan et al. (2016). The adult worms penetrate the horny layer of the gizzard, which may lead to a small dark red, ulcerated area. During penetration, they induce inflammation and cause damage to the muscular wall of the gizzard. They spent their entire life on the wall of the gizzard. Migrating larvae and adult worms traverse through the muscular wall, while the larvae and adults cause considerable damages. Damages of the muscular wall are directly proportional to the number of penetrating worms. We detected up to 100 worms from a single gizzard. Such heavily parasitized gizzards were found to be almost destroyed. Although Zenker’s necrosis, a glassy or waxy hyaline degeneration of skeletal muscle, was not detected, a curd-like caseous mass and liquefied materials were detected. Adult gizzard worms lay eggs in the lesions underneath the gizzard’s horny layer. Deposited eggs have been detected in the histological lesions of the affected gizzards. Eggs of helminths deposited in different organs play pivotal roles in developing pathological lesions. For example, eggs of schistosomes entrapped in different organs play critical roles in the development of schistosomiosis-related pathologies in various organs, resulting in hepatomegaly, splenomegaly, urinary bladder cancer and deformities in female genitalia (Anisuzzaman and Tsuji, 2020; Anisuzzaman et al., 2023). Similarly, acuariid eggs entrapped in the gizzard wall are also presumed to have significant roles in developing chronic pathological changes in the organs affected. On the other hand, if tissues are damaged, some ‘concealed self-antigens’ are released, known as damage-associated molecular patterns (DAMPs) or alarmins. Like pathogens-associated molecular patterns (PAMPs), DAMPs are also recognized by the pattern recognition receptors (PRRs) and elicit inflammation. We detected huge inflammatory cells surrounding the cross-section of parasites or parasitic eggs deposited. We detected numerous infiltrations of eosinophils. Infiltration of eosinophils has been detected in ectoparasitic and other helminth infections (Anisuzzaman et al., 2014, 2024; Anisuzzaman and Tsuji, 2020; Labony et al., 2022; Ritu et al., 2024). Eosinophils play vital roles in the entrapment and killing of helminths (Anisuzzaman et al., 2020).

We also noticed the thickening of the gizzard’s horny layer, and the layer loosened from the muscular layer of the gizzard. The lining of the gizzard is known as Koilin lining, which is made of a carbohydrate-protein complex rather than keratin. The Koilin membrane protects the muscular wall of the gizzard. Like other birds, chickens store their swallowed foods in the crop and then pass them down to the proventriculus, the true stomach. The proventriculus is glandular in nature and secrete gastric juice containing digestive enzymes. Then the gastric juice mixed foods enter the gizzard, which grinds the food. Thus, the gizzard causes mechanical degradation of food materials. The koilin membrane is vital for maintaining the soundness of the gizzard’s health and function, and helps digest food. If the gizzard becomes affected, then digestion will be significantly hampered.

We found that *C. hamulosa* survived well in serum (either FCS, DS or CS) supplemented DMEM or M199 medium, indicating a very high adaptability of the worm. The worm affects a large number of avian species, including chickens and ducks (Soulsby, 1982). Therefore, the worm’s survival for more than 7 days, with a higher viability score in the presence of DS or CS, is not surprising. In our previous studies on *Schistosoma mansoni* and *A. galli*, we found that serum harvested from definitive hosts greatly supports the survival and development of the parasites (Frahm et al., 2022; Ritu et al., 2024).

We found that IVM and LEV very efficiently killed all treated worms in a concentration-dependent manner. Both *ex vivo* and *in vivo* studies suggest that LEV and IVM are effective against several nematodes such as *Ascaris suum, A. galli, Haemonchus contortus*, and *Calilaria* (Dey et al., 2020; Parvin et al., 2023; Ritu et al., 2024; van de Weyer et al., 2024; Williams et al., 2024). LEV belongs to the group imidazothiazoles and is a levorotatory isomer of tetramisole, the main active component ofanthelmintic. It is highly effective against nematodes. Imidazothiazoles act as nicotinic acetylcholine receptor (nAChR) agonists. LEV binds to nAChRs distributed throughout the body wall. Blockage of nAChRs causes spastic paralysis of the worm, leading to the expulsion from the host. In addition, it also inhibits the enzyme fumarate reductase.

On the other hand, although the pharmaceutical dosage of ABZ and MBZ (10 mg/kg) is lower than that of LEV (25 − 50mg/kg), ABZ and MBZ did not kill the worms at the same concentration despite the extension of incubation time (48h), indicating the drugs are not effective enough to kill *C. hamulosa*. ABZ and MBZ belong to the benzimidazoles (BMZ) group of anthelmintics. The mode of action of BMZs, which has been elucidated, is very simple. Anthelmintics belonging to the group BMZ bind selectively to the β-tubulin of worms and inhibit microtubule formation. Due to the simplicity of the mode of action of BMZs, anthelmintic resistance (AhR) develop very rapidly against all BMZs. AhR to BMZ has already been detected, and the drugs are ineffective against *A. galli* and *Haemonchus contortus* (Dey et al., 2020; Parvin et al., 2022, 2023).

By conducting multiple trials using adult *C. hamulosa* isolated from different batches of chickens, we found that PPZ did not kill the worms even at 500 mg/ml concentration, indicating that PPZ is ineffective under *ex vivo* conditions, warranting further *in vivo* validation. PPZ is an old drug marketed in the early 20th century and is recommended for treating and controlling ascarids of mammals and birds. In Bangladesh, the drug is commonly used in chickens against *A. galli* without paraclinical assessment, and indiscriminate use of any anthelmintic for a long time leads to the development of AhR (Mitra et al., 2023). Anthelmintic resistance has recently been detected in *A. galli* infecting chickens (Ritu et al., 2024). The gizzard worms also cause co-infection with *A. galli* in chickens; thus, the development of AhR against PPZ in *C. hamulosa* is not surprising.

## Conclusions

The prevalence of *C. hamulosa* infection in ICs in Bangladesh is high. The present study indicated that the worm affects one-third of chickens reared in scavenging or semi-scavenging systems. This prevalence is influenced by the age of the chicken and seasons of the year. The worm induced gross and histological alterations on the muscular layer of the gizzard in affected chickens and destroyed the horny layer of the gizzard. Serum-supplemented M199 and DMEM media are effective in supporting the viability of *C. hamulosa* in *ex vivo* culture. LEV and IVM were highly effective against adult *C. hamulosa*, but ABZ, MBZ and PPZ have been found ineffective against the worm. This study provides data to help establish protocols to control and manage gizzard worms of poultry.

## Data Availability

Data supporting the findings of this study are available on request to authors.
